# Protecting my injured child: a qualitative study of parents’ experience of caring for a child with a displaced distal radius fracture

**DOI:** 10.1186/s12887-022-03340-z

**Published:** 2022-05-12

**Authors:** E. E. Phelps, E. Tutton, M. L. Costa, J. Achten, A. Moscrop, D. C. Perry

**Affiliations:** 1 Kadoorie, Oxford Trauma and Emergency Care, Nuffield Department of Orthopaedics, Rheumatology and Musculoskeletal Sciences, University of Oxford, Oxford, UK; 2 Oxford University Hospitals NHS Foundation Trust, John Radcliffe Hospital, Oxford, UK; 3 Parent Representative, Liverpool, UK

**Keywords:** Childhood fractures, Randomised controlled trial, Decision-making, Qualitative research, Interviews

## Abstract

**Background:**

Childhood fractures can have a significant impact on the daily lives of families affecting children’s normal activities and parent’s work. Wrist fractures are the most common childhood fracture. The more serious wrist fractures, that can look visibly bent, are often treated with surgery to realign the bones; but this may not be necessary as bent bones straighten in growing children. The children’s radius acute fracture fixation trial (CRAFFT) is a multicentre randomised trial of surgery versus a cast without surgery for displaced wrist fractures. Little is known about how families experience these wrist fractures and how they manage treatment uncertainty. This study aimed to understand families’ experience of this injury and what it is like to be asked to include their child in a clinical trial.

**Methods:**

Nineteen families (13 mothers, 7 fathers, 2 children) from across the UK participated in telephone interviews. Interviews were audio recorded, transcribed and analysed using reflexive thematic analysis.

**Results:**

Our findings highlight parents’ desire to be a good parent through the overarching theme “protecting my injured child”. To protect their child after injury, parents endeavoured to make the right decisions about treatment and provide comfort to their child but they experienced ongoing worry about their child’s recovery. Our findings show that parents felt responsible for the decision about their child’s treatment and their child’s recovery. They also reveal the extent to which parents worried about the look of their child’s wrist and their need for reassurance that the wrist was healing.

**Conclusion:**

Our findings show that protecting their child after injury can be challenging for parents who need support to make decisions about treatment and confidently facilitate their child’s recovery. They also highlight the importance of providing information about treatments, acknowledging parents’ concerns and their desire to do the right thing for their child, reassuring parents that their child’s wrist will heal and ensuring parents understand what to expect as their child recovers.

## Background

Childhood fractures are common with around one-third of children suffering at least one fracture before the age of 17 years old [1]. Childhood fractures have a significant effect on families altering children’s sleep, daily activities, independence and play. Parents can be required to miss work, change their household routines and experience concerns about how the fracture is healing and potential complications [2].

The wrist is the most common part of the body for children to break. The more serious wrist fractures (displaced distal radius fractures) often look visibly deformed. The children’s radius acute fracture fixation trial (CRAFFT) is a multicentre randomised non-inferiority trial of surgical reduction (surgery) versus casting without surgery (cast) for displaced distal radius fractures [3]. In the UK, these fractures are typically treated with surgery to realign the bones. The bones are sometimes held in position temporarily by wires (Kirschner wires or K-wires) or plates and screws. K-wires are stiff straight wires that pierce the skin, though protrude through the skin and are usually removed in outpatient clinics. However, surgery may not always be necessary in children as their bones are still growing. The growth of bones enables a process called ‘remodelling’ to occur. Remodelling allows deformity caused by fractures to self-correct as the bone grows.

Evidence regarding parent and children’s experience of orthopaedic injuries and treatment decision-making is limited. After a medial epicondyle fracture, parents’ endeavoured to make a decision about the best treatment for their child and facilitate their child’s recovery but they struggled to accept treatment uncertainty [4].

Recruiting children to clinical trials is challenging, particularly trials comparing operative vs non-operative treatments [5]. Parents can be uncomfortable making a decision on behalf of their child. They worry about the consequences of making the wrong decision, self-recrimination, unknown future complications and their child receiving the least effective treatment [6]. Parents’ moral obligation to be a good parent can exacerbate the challenge of making a decision about trial participation. Children are valued not just for the people that they are but also for the people that they will become [7]. Parents are therefore not only responsible for their child’s current wellbeing but also their future wellbeing. Parents desire to be a good parent and protect their child and their child’s future self as well as concerns about the appearance of their child’s wrist may influence their decision to participate in CRAFFT.

Building upon the work of Papiez et al. [4], this study sought to understand families’ experience of a traumatic orthopaedic injury where there may be concerns about the appearance of the wrist. This study explored parents’ experience of their child’s injury, treatment, decision-making and the early phase of recovery.

## Methods

The CRAFFT study is registered with the International Standard Randomised Controlled Trials Number Registry (ISRCTN10931294: 27/02/2020). Recruitment is ongoing in 47 NHS sites. As of March 2022, 348 families had consented to CRAFFT. One-hundred-and-sixty-three families declined participation including 43 preferring a cast and 76 preferring surgery.

### Sample and recruitment

A purposive sample of parents who were approached about participating in CRAFFT were invited to take part in an interview. The sample aimed to include variation in the children’s age, gender, severity of injury, treatment, the hospital they attended, and decision to take part in CRAFFT. As part of the initial consent process for CRAFFT, families were informed of the qualitative study and asked whether they could be approached about taking part. Parents who were willing to be approached provided electronic consent to be contacted and contact details. Parents were emailed an information sheet about the qualitative study and were contacted by telephone to answer any questions and, if they wanted to take part, arrange a time for the interview. Parents underwent a separate informed consent discussion. Verbal informed consent was recorded and witnessed by an administrator who had undertaken research integrity training referred to as Good Clinical Practice (GCP).

### Interviews

The methodology for the interviews drew upon Heideggerian phenomenology and notions of Dasein (being or presence) [8]. This enabled exploration of participant’s experience of what it is like to be in their lifeworld. It included their personal and social life in the context of temporality, the past, present and future. The researcher acted by focusing their ‘phenomenological gaze’ on the participant and from this interaction developed an understanding of their feelings, thoughts and relationships [9]. This has proved useful in studies of injury in adults particularly where there is limited evidence of patient experience, as in this study [10, 11]. Interviews were by telephone up to 3 months post injury and lasted up to an hour. An experienced female qualitative researcher with a PhD, a background in psychology, prior research experience of injury in children and who is a parent conducted the interviews. The researcher did not know the participants prior to the study and one parent chose not to take part when approached. Children who wanted to take part joined their parent. Participants were offered a copy of their transcript, though none took up this offer.

### Patient and public involvement (PPI)

Patient and public involvement was undertaken in four main ways; i) a parent and young person PPI group were involved in the design of the study, study information and explainer videos, ii) two in-depth PPI interviews were conducted with parents of children with a displaced distal radial fracture to sensitise the researchers to parent’s experience of this injury, iii) two parents were PPI representatives on the management and steering committee and were involved in all study decisions, iv) one PPI parent is a co-author and worked with the researchers to develop this article.

Interviews explored: i) what injury, treatment and early recovery is like for families, and ii) parents’ experience of being asked to include their child in CRAFFT. Open questions allowed parents to describe their experience in their own words with prompts used to gain insight into how they felt. Children who took part in the interview had the option of talking to the interviewer at the start of their parents’ interview or throughout the interview. Child interview questions included: i) what were you doing when you hurt your wrist? ii) what was it like being in the hospital? iii) how does your wrist feel now?

### Analysis

Interviews were audio recorded, transcribed and managed using NVIVO 11 (QRS Warrington). An experienced qualitative researcher led the analysis, using a reflexive approach to thematic analysis. This inductive approach acknowledged the active role of the researcher in interpreting patterns of meaning within the data [12]. To gain an understanding of each participant’s world, analysis developed through listening to the recordings, reading the transcripts and writing field notes [9]. Field notes were used to record initial thoughts and important elements of experience. Analysis was iterative, data was coded and new codes added as interviews were undertaken. Similar codes were grouped to form categories and then into themes that identified the structure of experience [9]. Two experienced qualitative researchers (EP and ET) discussed the data throughout analysis and further reflexive discussions took place with the co-authors of this article.

Rigour and trustworthiness [13] were achieved through immersion in the world of the participants, reflecting on the developing theoretical framework and the research team’s positionality, the inclusion of extracts of data to illustrate interpretations and descriptions of the participants, context and methods to enable transferability of findings. Data saturation was achieved. The consolidated criteria for reporting qualitative research (COREQ) guidelines informed this article.

## Results

### Participants

Nineteen families (13 mothers, 7 fathers, 2 children) participated in a telephone interview between November 2020 and May 2021. This included 15 individual parent interviews, two parent-child dyads and one parent dyad. Families were recruited from eight hospitals. Eighteen families participated in CRAFFT. Nine children received surgery (followed by a cast) and 10 received a cast. Six children (3 who received surgery) had a completely offended fracture (the most severe fracture). Children were between four and 10 years old (mean age 7.3). Five children were girls and 14 were boys.

### Findings

**Fig. 1:**
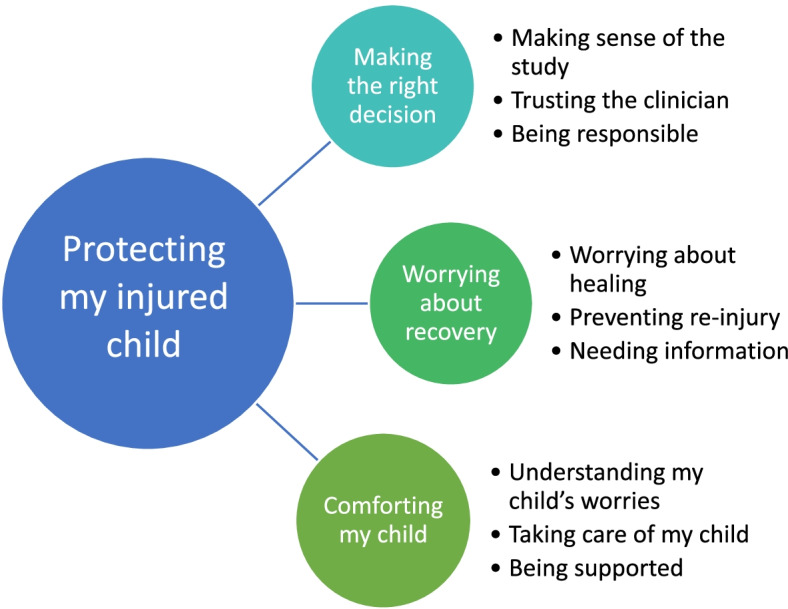
Protecting my injured child - themes and categories

The study identify the overarching theme “protecting my injured child”. To protect their injured child, parents endeavour to make the right decisions about treatment and comfort their child but they worry about their child’s recovery. Figure 1 presents the three themes and nine categories within protecting my injured child.

#### Theme 1: making the right decision

To make a decision about participation, parents endeavoured to make sense of the information they received about their child’s injury, the treatment options and their risks. Parents questioned clinicians as they strived to make the right decision, feeling responsible for the outcome of their child’s injury.

### Making sense of the study

For the majority of parents the study made sense; they understood surgery was the standard treatment for this fracture in the UK but there is evidence to suggest a cast can lead to equally good outcomes as children bones are still growing. However, they could struggle to take in information due to time pressures, the busy ED environment, distress at seeing their child injured and their focus on comforting their child. Parents could feel pressured, needing more time to make the decision and discuss the options with others.

*“You’re in A&E, it’s so noisy and there’s people coming and going all the time and you’re trying to comfort (your child) and read what’s going on.”* (Interview#19, Mother of a 6-year-old child)

Parents felt CRAFFT was introduced before they knew enough about their child’s injury. They wanted more information including statistics and pictures of children whose wrists have healed in a cast.

*“Almost the first thing that was mentioned was the CRAFFT study and she (my wife) felt that got in the way of clinical advice. I’m not saying the problem wasn’t described at all and the treatments weren’t set out but the CRAFFT study and whether or not we wanted to take part in it came really early on in the conversation.”* (Interview#14, Father of a 10-year-old child)

Parents were reassured about taking part when clinicians explained the long-term outcomes were similar. However, they tended to have a treatment preference. Preferences evolved while parents considered the risks and benefits or were based on “gut feelings” about which treatment was best.

*“I felt very pressured to make a decision within a certain amount of time and I didn’t have all the information, I just went with my gut that’s all.”* (Interview#17, Mother of a 4-year-old child)

Parents who preferred casting worried about the trauma of an operation, anaesthetic, infection, and staying in hospital. Several families participated in CRAFFT for a chance to avoid surgery.

*“It kind of made sense to me that a child’s bone would be able to heal itself and that the surgery sounded more of an aggressive fix to the problem really and I was a bit nervous about my child going for an operation anyway.”* (Interview#18, Mother of a 6-year-old child)

Those preferring surgery trusted the standard treatment perceiving surgery to guarantee their child’s wrist would heal and would ‘be straight’ (Interview#10, Father of a 5-year-old child). Parents needed reassurance that if their child was treated with the cast their wrist would not look broken forever, there were alternatives should the wrist not straighten, and their child would not experience more pain or a slower return to sport.

*“The doctor was quite reassuring and saying that if it didn’t work then there were other things that they could do and that they could still fix it. Her arm wouldn’t be bent forever.”* (Interview#13, Mother of an 8-year-old child)

### Trust in the clinician

Parents wanted to ensure their child would not be disadvantaged by participating in CRAFFT. Parents trusted staff but sought reassurance that their child’s wrist would heal. They questioned clinicians to confirm the cast was appropriate for their child as it could be hard to envisage the arm straightening without surgery.

*“I guess it’s quite a leap of faith isn’t it in terms of the doctors are saying this but is that true? Personally, I felt they knew more than me.”* (Interview#6, Father of a 4-year-old child)

Parents perceived staff as open and honest, sharing the available evidence, answering questions and explaining they would include their own child.

*“They took their time to explain everything, the research and the evidence that they’ve got.”* (Interview#9, Father of a 10-year-old child)

However, some parents sensed that staff withheld information, felt clinicians were unable to talk freely or say what they would do if it were their child and this could frustrate parents.

*“That’s when I said, what would you do, and he said I can’t really answer that because it’s your decision.”* (Interview#10, Father of a 5-year-old child)

Parents trusted staff would not offer the trial if it could harm their child.

*“I then asked some questions about if they were sure that it didn’t need surgery... she said no absolutely not and they wouldn’t have suggested the CRAFFT study if surgery was necessary.”* (Interview#19, Mother of a 6-year-old child)

### Being responsible

Parents felt responsible for the decision to include their child in a study and for the outcome of their child’s injury. The majority of parents were content with their decision but some parents experienced worry and regret. Family decision-making was important but could be challenging with hospitals only allowing one parent to accompany children owing to restrictions related to the COVID pandemic. Some clinicians facilitated family decision-making by giving families time to discuss the study at home, or by enabling both parents to speak to the clinician via video conference. Children were often involved in the decision with some children wanting to take part for a chance to avoid surgery.

*“She did actually say ‘I think it would be a good thing to be in the trial’ and so she was quite happy. So yes, we kind of made the decision together.”* (Interview#7, Mother of a 10-year-old child)

Parents did not always share the same view. At times, one parent made the decision they felt was right, consenting to CRAFFT despite suspecting that their partner may not agree. Making the decision on their own could be burdensome, ‘I would be the one blamed for it’ (Interview#11, Father of a 7-year-old child). They did not want to be responsible for a decision that harmed their child, left them with a ‘wonky arm’ or needing further surgery.

*“Having to potentially bring him back in at some stage to re-break and reset the bones or for him to have to go potentially for the rest of his life with a big bend in his forearm. I just didn’t feel I could make that decision for him because I don’t know what impact that could potentially have on him for the rest of his life.”* (Interview#15, Mother of an 8-year-old child)

Parents could be relieved their child was randomised to surgery even if it was not their initial preference. Surgery guaranteed their child’s wrist was fixed and it was felt to be the right treatment for their child. They subsequently questioned the non-surgical treatment.

*“Once I did know she was having the surgery I felt more relieved. I thought she needs the surgery and at least we’re coming out at the end of the day with a straight arm. On reflection I don’t think she would have coped that well if she’d have just been in the cast… she’s been very conscious the whole time of how it looks and how it’s going to look when the cast comes off.”* (Interview#7, Mother of a 10-year-old child)

Parents could regret their decision to take part in CRAFFT. One parent felt their child had not benefitted from surgery as their wrist was not straight. Another parent felt uninformed and under pressure when making the decision.

*“I think if I did it again I’d want a cast rather than surgery and we’ve come to where we thought we’d be with the cast anyway, and we’d be better off not having the surgery in the first place. We still had a lump afterwards anyway.”* (Intervirew#10, Father of a 5-year-old child)

#### Theme 2: worrying about recovery

Parents worried about how their child’s wrist was healing, some encouraged their child to use their wrist while others restricted activities to prevent further injury. Parents needed more information about recovery to help their child and reassurance from clinicians.

### Worrying about healing

Parents worried their child’s wrist looked “deformed” after their cast was removed. Seeing their child’s arm was still “wonky” could make parents nervous about how it was healing and how strong it was. Some parents were disappointed their child’s wrist had not straightened as much as they had expected. Parents in both treatment groups did not expect stiffness or pain when the cast was removed and were worried their child could not use their wrist as before. Children were often protective of their wrist and reluctant to use it initially. Some children needed encouragement from parents to resume activities.

*“I was a little bit shocked to begin with because she didn’t seem to be able to move her wrist as well as the other wrist… I think she felt quite protective of her arm when it first came out. She wouldn’t let me put her coat on and things like that.”* (Interview#13, Mother of an 8-year-old child)

### Preventing re-injury

Parents worried about their child re-injuring their wrist or breaking another bone. Several parents described their child as active or fearless and this contributed to their worry. Parents felt their child’s wrist was unprotected without the cast and tried to ensure their child understood the need to be careful. They were often more worried than their child about resuming activities, limiting their child’s activity to prevent further injury.

*“I was very worried that if he fell over he would have another hand broken, leg broken or he would have no teeth. I was going behind him saying ‘calm down, can you please stop’ but as I said he is very active.”* (Interview#12, Mother of a 5-year-old child)

### Needing information

Parents were shocked by the lack of support once the cast was removed and felt uncertain about their child’s recovery. They wanted confirmation that their child’s wrist was healing.

*“We had an x-ray and they said that the broken wrist had actually recovered or healed by 90% and said that it was strong enough to keep the cast off.”* (Father#9, Father of a 10-year-old child)

Parents wanted more information about what their child should or should not do once the cast was removed. Some parents tried to find information on the internet but this could lead to concern.

*“He’s asking questions which my wife and I aren’t sure about and had to read on the internet about it…sometimes you will go and search for something very little and it will bring up something that will make you worried.”* (Father#11, Father of a 7-year-old child)

Parents did not always know what to ask especially if this was their first experience of a fracture. They could feel unprepared and felt they had not asked enough questions when they had the opportunity.

*“Just so that we could have been prepared really. I felt as if I wasn’t quite prepared, I felt a little bit on the back foot so to speak.”* (Interview#7, Mother of a 10-year-old child)

Others felt they had received all the information they needed, drew upon prior experience of injuries, or felt confident to ask questions. Knowing they could approach staff for more information and the confidence to do so enabled parents to access more support if needed. Parents observed their child using their wrist, noting concerns to ask at follow-up appointments or contacting clinicians when they became concerned.

#### Theme 3: comforting my child

Parents took into account their child’s worries and found ways to take care of their child. They valued support from caring clinical staff who put their child at ease.

### Understanding my child’s worries

Injuring their wrist and visiting hospital could be traumatic for families. Prioritising their child’s comfort and alleviating their child’s worries was important. Children could be tired, scared or in pain.

*“It got to about 2 o’clock in the morning and we were sat on plastic chairs in the A&E waiting room. He had had no sleep, and he’d had nothing to eat since lunchtime at school that day then he started to get very weepy. He just wanted to get home and for it to be over.”* (Interview#15, Mother of an 8-year-old child)

Children were conscious of the look of their wrist, asking when their wrist would look normal again, commenting that their wrist was wonky or were upset to have scars from K-wires. Parents tried to reassure their child that their wrist would straighten and their scars would fade with time.

*“Sometimes he’ll say ‘it still looks a bit wonky doesn’t it?’ but I just explain to him that it’s going to be like that for a while yet but it just means the bones are still healing but it’s just the way your arm is but it will get better.”* (Mother#5, Mother of a 5-year-old child)

Knowing what was important to their child and their prior experiences enabled parents to understand their child’s concerns. Some children worried about the look of their wrist, while other children were more concerned about returning to sports.

*“I think the key for my son…the longer term of it straightening up was not really a problem - as long as he can play football, ride his bike and do those sorts of things.”* (Mother#3, Mother of a 10-year-old child)

Parents also understood that prior hospital experiences could lead children to worry.

*“He had to have an anaesthetic two years ago for his appendix. I don’t think he enjoyed the whole experience of being in hospital for a week and so he was a bit nervous about being in hospital again.”* (Mother#18, Mother of a 6-year-old child)

### Taking care of my child

Parents endeavoured to take care of their child, taking into account their child’s personality and what they could cope with to make the experience bearable.

They found ways to help their child cope with their injury and the hospital environment, for example protecting them from too much information or noise.

*“As we were coming out of the hospital he started getting really upset and he just said I’m really scared and I don’t’ want to get the cast off now. When we went back, I took ear defenders with us and I told the nurse that he’d got scared last time because he was watching someone have theirs (cast) removed.”* (Interview#5, Mother of a 5-year-old child)

Children often needed help with everyday tasks while in the cast such as dressing, toileting and cutting food.

*“I had to help him with virtually everything, he can’t get himself dressed, he can’t put toothpaste on his toothbrush and I had to help him with his toilet.”* (Interview#15, Mother of an 8-year-old child)

Children could also require a lot of encouragement to feel confident to undertake activities.

*“He’s the kind of boy who overthinks quite a lot. There’s always a million questions and always worrying. So there was a lot of talk and a lot of trying to calm him down. The doctor said he could have a shower three days after removing the cast. It took us about eight days for us to convince him that he could have a shower”.* (Interview#11, Father of a 7-year-old child)

### Being supported

Parents needed support, reassurance and information from staff to help them take care of their child. They were grateful for the care and support they received. Reassurance from staff provided comfort to parents, giving them confidence that their child would be cared for. Parents appreciated staff acknowledging their worries particularly their concern about how their child’s wrist was healing.

*“Hopefully it will go back and get back to being straight again. When I spoke to the doctor he was very confident that it would actually go and so that made me feel more at ease at how confident he was.”* (Interview#13, Mother of an 8-year-old child)

Parents valued being involved in their child’s care and the support they received to enable this. This included being involved in decisions about treatment, cast removal and the use of gas and air.

*“I just think that the doctors have been really proactive in giving me information and letting me make decisions but also helping me to understand things more.”* (Interview #18, Mother of a 6-year-old child)

Parents valued staff putting their child at ease and answering their child’s questions. Children wanted to know what type of cast they would have, what the operation would feel like, and what their arm would look like.

*“[They were] very good in doing that and so in some ways he seemed excited almost when we went back for the operation. He was really at ease and ready for what was ahead I think because of how people were communicating with him”.* (Interview#6, Father of a 4-year-old child)

If their concerns were dismissed parents could feel unsupported.

*“I felt it was very, I won’t say ‘condescending’, but he (the consultant) made it sound like the only people really worried about it were grandparents and parents. He said they put too much emphasis on what the arm looks like.”* (Interview#17, Mother of a 4-year-old child)

## Discussion

Our findings show that when confronted with injury and the potential for trial participation, being a good parent and protecting their child underpinned parents’ experience of making the right decision, comforting their child and worrying about recovery. Families sought to make sense of information provided in challenging circumstances and hoped they had made the best decision for their child. The implications for practice are that there needs to be: i) recognition of the high degree of challenge and emotional impact involved in trial participation, ii) support for recovery from fractures to help alleviate the degree of worry, and iii) family centred practices that support parents to nurture their child through treatment and maximise their potential for recovery. In the specific context of the CRAFFT study, decision making in an emergency setting, uncertainty about bone remodelling and worries about ongoing deformity, alongside the lack of knowledge and experience of fracture treatment and recovery, were key sources of concern.

The overarching theme of ‘protecting my injured child’ supports and extends existing research on children with traumatic elbow fractures [4]. Parents wanted the best for their child, felt responsible for the decision to enrol their child in the trial and felt responsible for their child’s subsequent recovery. They were fearful of making the wrong decision and living with regret, as noted in research in other specialities [14, 15]. Other randomised controlled trials in paediatrics have identified that parents felt ‘protected’ from feeling responsible for a poor outcome by randomisation [16], which is a strategy that could be explored within the CRAFFT study.

Decision making in an emergency situation was a challenge for parents, particularly when faced with the fast pace and busy nature of the emergency environment, often where only one parent could accompany the child. Parents were distressed by their child’s injury and were focused on comforting their child. Absorbing information and making decisions within the time constraints was a challenge. Parents who were given the opportunity to consider the study overnight found time helped. Parents who were asked to make a decision quickly felt pressured or wanted a decision to be made and treatment initiated. The optimal time for decision making in the emergency setting is hard to determine. A trial amongst children with appendicitis identified 1–2 hours was the optimal study deliberation time, though noted that parents were apprehensive about treatment delays as a consequence of study participation [5].

Uncertainty about bone remodelling and worry about deformity exacerbated parents’ concerns about participation in this trial. Parents wanted certainty that their child’s arm would straighten and not be deformed. They trusted surgeons, but also questioned the evidence or sought reassurance about subsequent treatments if the arm were not to straighten. Parents struggled with what they perceived was a severe injury and the stark contrast they faced between surgical and non-surgical treatment. Other studies amongst adults taking part in surgical trials identify participants often struggle to make a decision regarding trial inclusion, which is more marked when the injury is severe or there is a substantial difference between the treatments [17, 18]. In this study parents believed that surgery would guarantee that their child’s arm would straighten. However, irrespective of whether the wrist was treated with surgery or non-surgical treatment, parents had ongoing worries about the appearance of the wrist, with parents feeling responsible for the cosmesis in both groups. Deformity and the stigma of appearing different can generate strong emotions such as guilt, stress, anger and anxiety in parents of children with a condition or injury as they strive to protect their child [19]. This can lead parents to pursue surgical treatments to normalise their child’s appearance [20]. Our study suggests that further research is needed to explore parent’s experience of deformity during recovery from wrist fractures, to understand how families cope over time.

Parents lacked knowledge and experience of a fracture, and often needed help to feel confident in facilitating their child’s treatment and recovery. Communication that parents felt was ‘supportive’ recognised the emotional impact of injury and the challenge of decision making in an emergency setting. It may also help them cope with the anxiety generated by having an unwell or injured child [21]. Parents nurtured their child towards recovery, but worried about pain, stiffness, appearance, further injury and return to activities. Feeling uncertain is known to contribute to worry and anxiety [22]. Furthermore, children can observe and mirror their parents’ emotional responses, which may influence the child’s coping strategies. Parents’ emotional response to injury may therefore impact upon children’s experience of surgery and recovery [23]. Knowing more may help parents to worry less and develop good strategies to support recovery. Advice about what to expect as their child recovers, how their child can use their wrist and when to resume activities could help parents to feel more confident in facilitating recovery. Involving children throughout recovery is also important, as it has been shown that they can feel excluded from discussions about pain, feel their parents often do not know when they are in pain and that pain limits their activities [24]. Further research is needed to determine the information and support required to increase parents’ confidence to nurture their child during recovery, while engaging and listening to children during this process.

### Strengths and limitations

This study included mothers and fathers from across the UK and parents of children who received both study interventions. Only one parent who declined participation in the CRAFFT trial was interviewed, though we recognise that those who decline participation may have different views and experiences to those who participated in the study. Two children joined their parents for the interview. Further interviews with children would give them the opportunity to voice their own experience, rather than relying on their parents’ interpretation. This may aid understanding of what is important to children after injury.

## Conclusions

This study reveals the challenges that parents endure to protect their child after injury, which is amplified with the introduction of a research study. We highlight the particular difficulties of undertaking trials in children’s trauma, with the urgency of the condition and the limit on time for contemplation adding a further challenge for parents. However, ‘randomisation’ could be seen as a vehicle to ‘protect’ parents from making the wrong decision, in the face of clinical uncertainty. A parent’s worry for their child is ongoing throughout recovery, despite often feeling they do not know enough. Good communication from clinicians may help parents feel confident by acknowledging their concerns, recognising parents’ struggle to make the right decision, reassuring parents that their child’s wrist will heal and by providing more clarity about what they can expect while their child recovers.

## Data Availability

We do not have consent for storage in a repository. For further information, contact the corresponding author.
